# In vitro singlet state and zero-quantum encoded magnetic resonance spectroscopy: Illustration with N-acetyl-aspartate

**DOI:** 10.1371/journal.pone.0239982

**Published:** 2020-10-01

**Authors:** Andrey N. Pravdivtsev, Frank D. Sönnichsen, Jan-Bernd Hövener

**Affiliations:** 1 Section Biomedical Imaging, Molecular Imaging North Competence Center (MOIN CC), Department of Radiology and Neuroradiology, University Medical Center Kiel, Kiel University, Kiel, Germany; 2 Otto Diels Institute for Organic Chemistry, Kiel University, Kiel, Germany; Rijksuniversiteit Groningen, NETHERLANDS

## Abstract

Magnetic resonance spectroscopy (MRS) allows the analysis of biochemical processes non-invasively and in vivo. Still, its application in clinical diagnostics is rare. Routine MRS is limited to spatial, chemical and temporal resolutions of cubic centimetres, mM and minutes. In fact, the signal of many metabolites is strong enough for detection, but the resonances significantly overlap, exacerbating identification and quantification. Besides, the signals of water and lipids are much stronger and dominate the entire spectrum. To suppress the background and isolate selected signals, usually, relaxation times, J-coupling and chemical shifts are used. Here, we propose methods to isolate the signals of selected molecular groups within endogenous metabolites by using long-lived spin states (LLS). We exemplify the method by preparing the LLSs of coupled protons in the endogenous molecules N-acetyl-L-aspartic acid (NAA). First, we store polarization in long-lived, double spin states, followed by saturation pulses before the spin order is converted back to observable magnetization or double quantum filters to suppress background signals. We show that LLS and zero-quantum coherences can be used to selectively prepare and measure the signals of chosen metabolites or drugs in the presence of water, inhomogeneous field and highly concentrated fatty solutions. The strong suppression of unwanted signals achieved allowed us to measure pH as a function of chemical shift difference.

## 1. Introduction

Magnetic resonance (MR) has found a multitude of applications in medical imaging, from anatomy to motion, function and metabolism [1–3]. Likely, one of the most promising, yet least delivering applications is in vivo MR spectroscopy (MRS). MRS provides a non-invasive window into the biochemistry of living organisms–basically a virtual biopsy. Alas, it does not live up to this promise, as MRS is rarely used in routine diagnostics.

In the brain diagnostics, the success of MRS is hampered by the interplay of two major issues: first, a comparatively weak signal of the metabolites of interest, and second, confounding overlapping resonances and strong background signals mostly from water, lipids and proteins [4]. As a result, most of the time, only highly concentrated metabolites are detected, such as the major brain metabolites N-acetyl-l-aspartic acid (NAA), creatine (Cr), choline (Cho), myo-Inositol (Myo), lactate, alanine and non-exchangeable protons on proteins. For example, up to 0.1% of the brain tissue wet weight of mammals belongs to NAA [5] which is associated with neuronal integrity [6, 7].

^2^H-enriched, nonradioactive molecules like ^2^H-glucose are used to obtain background-free spectra in vivo. Valuable insights into biochemistry were gained by this approach [8–10]. However, because of the limited amount of substrate that can be administered in conjunction with the low magnetogyric ratio of ^2^H, the signal remains low and does not allow routine high-resolution imaging.

Hyperpolarization techniques boost the signal of selected, isotopically labelled metabolites. This way, the fate of a dedicated, polarized agent can be followed in vivo with increased spatial, chemical and temporal resolution [11–13]. For example, hyperpolarization allows imaging the distribution of the hyperpolarized agent, mapping of tissue pH or metabolic conversion [14]. These methods provide different information than conventional MRS without injections, where a quasi-steady state of the metabolism is measured.

In humans, hyperpolarization has shown great promise for imaging prostate cancer, brain cancer, monitoring therapy response, heart metabolism and lung imaging [15–20]. Still, the method is limited by the relatively short lifetime of the signal enhancement and thus short observation window, as well as the limited amount of hyperpolarized agent that can be injected. Additional requirements include a hyperpolarizer, specialized imaging sequences and an X-nuclei channel for the MR system [21–24].

One may argue that the signals of many metabolites are already strong enough for many applications without further enhancement. Unfortunately, it is difficult to isolate the signal of an individual metabolite in the densely packed ^1^H spectrum of the human brain; the resonances of many interesting metabolites overlap or differ only by a few parts per million. Thus, much inventiveness has gone into the development of suppression and spectral editing techniques [7, 25–32], exploiting differences in chemical shift, relaxation times or J-couplings.

The presence of numerous small molecules, proteins and fat greatly complicates the analysis of a spectrum; all aliphatic protons abundant in fat and small molecules occupy the same chemical shift region of 1–5 ppm. Therefore, usually, only the sharpest singlets or doublet signals of CH_3_ protons of small molecules are prominent in ^1^H-MRS [1, 7, 27, 28].

The use of so-called singlet or long-lived states to improve MRI was suggested before. Often, the singlet state of strongly coupled nuclei is much longer lived (T_LLS_) than the corresponding longitudinal magnetization (T_1_) [33–35]. It was suggested to use such long-lived states (LLS) as a new MR contrast [31, 32, 36], to measure slow diffusion [37, 38] or to track chemical exchange [39]. But the long lifetime is not the single unique property of LLSs. Just as interesting is that broad-band decoupling or continuous-wave excitation (CW) preserve these states and even prolong their lifetime [40], while “normal” resonances are saturated. To get more information from the spectra, the “suppression of (the signal of) undesired chemicals using contrast-enhancing singlet states” (SUCCESS) was proposed by DeVience et al. [41]. During the publication process of this paper, Glöggler et al. [31] proposed a new method for filtering singlet states. Thus far, however, LLS-filtering methods were demonstrated only on high-resolution NMR devices, often with strong radio frequency (RF) power applied.

**Fig 1:**
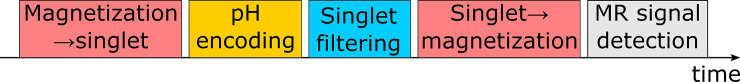
Suggested 5-step SISTEM pulse sequence: (1) magnetization of ^1^H nuclei is transferred to LLSs and zero-quantum coherences; (2) encoding a feature (here: Chemical shift difference that can be correlated with the pH value, Figs 4 and 5); (3) background suppression: Only singlet spin states (or zero-quantum coherences) pass through the filter; (4) conversion of singlet state into observable magnetization and (5), MR signal detection.

**Fig 2:**
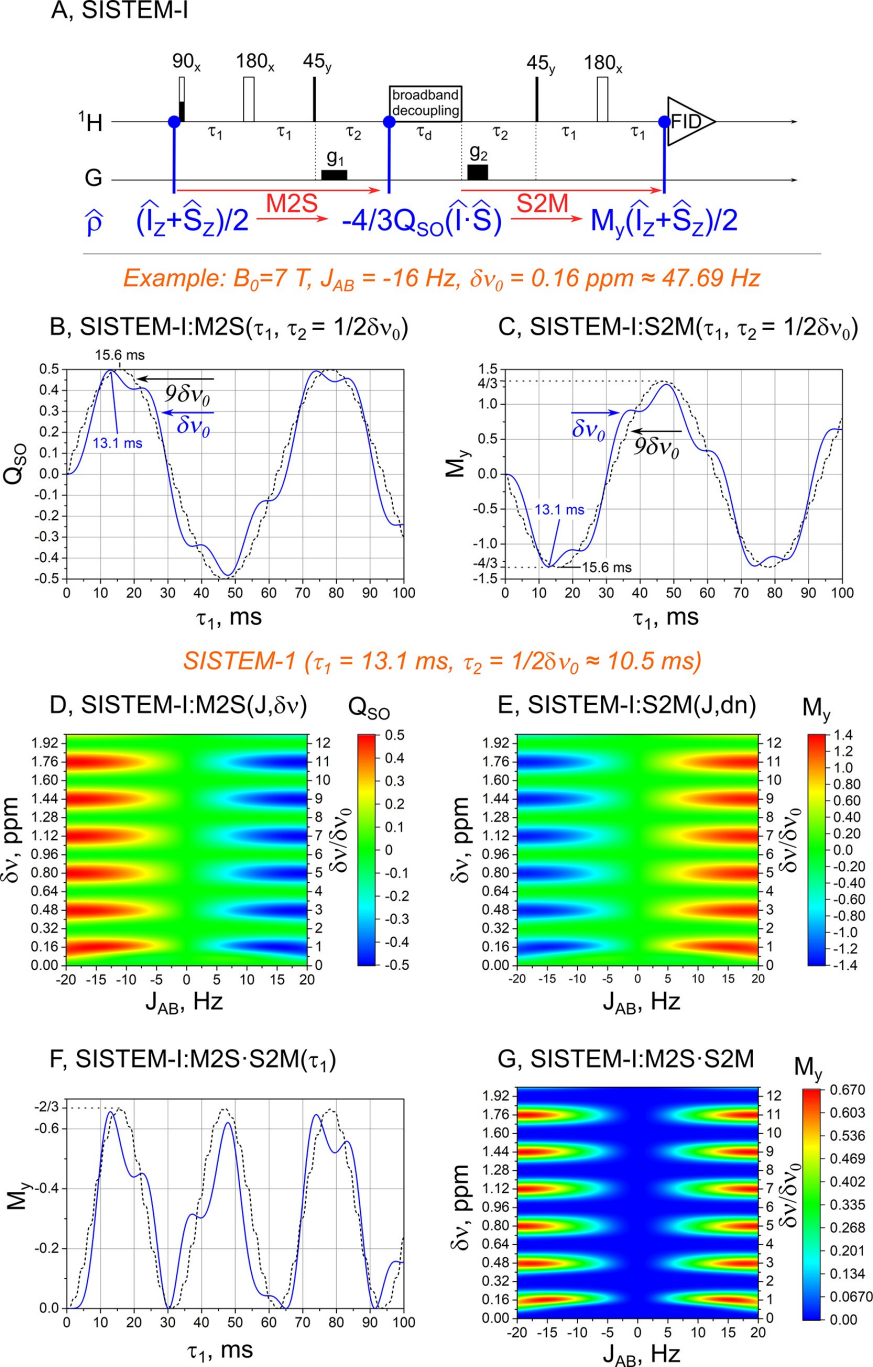
Scheme of SISTEM-I (A), its magnetization-to-singlet (M2S) and singlet-to-magnetization (S2M) polarization transfer performance (B-E), and efficacy of the entire sequence (M2S·S2M, F, G). For the AB-type two-spin system investigated (*δν*_0_ = 0.16 ppm, *J*_*AB*_ = -16 Hz, θ≅9° at *B*_0_ = 7 T, dashed lines in B,C,F), M2S and S2M polarization transfer deviate from the sine-shape, sin(2*πτ*_1_*J*_*AB*_), predicted for a weakly coupled AX-type two-spin system (here, *δν* = 9∙*δν*_0_, *J*_*AB*_ = -16 Hz, θ ≅1°, solid lines in B,C,F). [39] Optimal parameters for the AB system are *τ*_1_ = 13.1 *ms* and *τ*_2_ = 1/Δ*v*_0_. In these conditions, “M2S” element provide conversion of 100% magnetization to 50% of singlet-triplet imbalance ($\frac{1}{2}({\hat{I}}_{z}+{\hat{S}}_{z})\overset{"M2S"}{\to }-\frac{4}{3}(\hat{I}∙\hat{S})∙Q_{ST}$, with Q_ST_ = ½), (D). “S2M” polarization transfer element demonstrates similar behavior (E), here calculation is started with Q_ST_ = 100% (($-\frac{4}{3}(\hat{I}∙\hat{S})\overset{"S2M"}{\to }\frac{1}{2}({\hat{I}}_{y}+{\hat{S}}_{y})∙M_{y}$, with *M*_*y*_ = 4/3)). The maximum signal after SISTEM-I sequence is 2/3 of the initial magnetization ($\frac{1}{2}({\hat{I}}_{z}+{\hat{S}}_{z})\overset{"M2S∙S2M"}{\to }\frac{1}{3}({\hat{I}}_{y}+{\hat{S}}_{y})$, F,G). The performance of the sequence is high for the two spin-½ systems with |*J*| = 16±4 Hz and chemical shift difference of (2*n*−1)(*δν*+10%) where n is natural number (G). *τ*_2_—intervals can be substituted by Thrippleton–Keeler zero-quantum filter [39, 47] to make the sequence independent of the chemical shift difference.

We suggest to use “singlet-state encoded MR” (SISTEM) as a more general term than SUCCESS and because it reflects the physics of the sequence better than the name of an author (e.g. “Sarkar-II” [39]). Using this terminology, in this paper, we study the properties of SISTEM sequences (**Fig 1**), namely SISTEM-I (former Sarkar-II, **Fig 2A**) and SISTEM-II, a new sequence shown in **Fig 4A**. We selectively prepared the signal of specific, endogenous metabolites using SISTEM at high-resolution NMR and small-animal MRI unit. SISTEM sequences are modular, broad-band and can require only little RF power deposition. We show that this method may serve purposes beyond background suppression e.g. for pH imaging.

## 2. Materials and methods

### 2.1. Chemistry

N-acetyl-l-aspartic acid (NAA, Sigma-Aldrich, 00920, CAS: 997-55-7, Scheme 1), DL-lactic acid (Sigma-Aldrich, 69785, CAS: 50-21-5), l-alanine (Sigma-Aldrich, A7469, CAS: 56-41-7), creatine monohydrate (Sigma-Aldrich, C3630, CAS: 6020-87-7), choline chloride (Sigma-Aldrich, C7017, CAS: 67-48-1), l-glutamic acid (Sigma-Aldrich, 49449, CAS: 56-86-0), myo-Inositol (Sigma-Aldrich, I7508, CAS: 87-89-8) were purchased and used without further purification.

#### Model solution 1 (MS1)

Each of the substrates listed above was dissolved in D_2_O (Deutero GmbH, 00506) to yield a concentration of 10 mmol/L. pH was adjusted to the desired value by adding NaOD (Deutero GmbH, 03703) or DCl (Sigma Aldrich, 543047); pH-dependent NMR spectra of all substrates are given in **SM**. Note that only NAA is discussed in the main text.

#### Model solution 2 (MS2)

300 μL D_2_O solution of 10 mmol/L of NAA was mixed with 300 μL food-grade dairy cream (30% fat concentration).

#### Model solution 3 (MS3)

1500 μL deionized H_2_O solution of 100 mmol/L of NAA with a pH 5.15.

### 2.2. NMR and MRI

All high-resolution NMR spectra were acquired on a 600 MHz vertical bore NMR spectrometer (Bruker Avance II) with a cryogenically cooled probe (TCI) and 5 mm NMR tubes (Wilmad).

Nonlocalized spectra were acquired with a 7 T horizontal bore MRI with a 30 cm diameter of the inner bore (Bruker 7/30 ClinScan), equipped with a ^1^H transmit-receive volume coil with an inner diameter of 8 cm for excitation and a single loop surface coil with an inner diameter of 0.9 cm for detection [42]. **MS3** was filled into a 1.5 mL container (Eppendorf vial) and placed in the pick-up coil at the isocenter of the magnet. Both NMR and MRI were adjusted using the standard procedures for resonance frequency, shim, flip angle and receiver gain.

Routine nonlocalized spectroscopy and inversion recovery sequences were used; SISTEM-I and -II were implemented using the manufacturer's software (TopSpin 3.2 or Siemens VB15, IDEA 1.5b1.63). The same software was used to process data.

The signals were quantified by integrating amplitude spectra (**Fig 4, S2 Fig in S1 File**).

### 2.3. Pulse sequence

In general, SISTEM is composed of five steps with different functions (**Fig 1**), some of which may occur at the same time.

**Fig 3:**
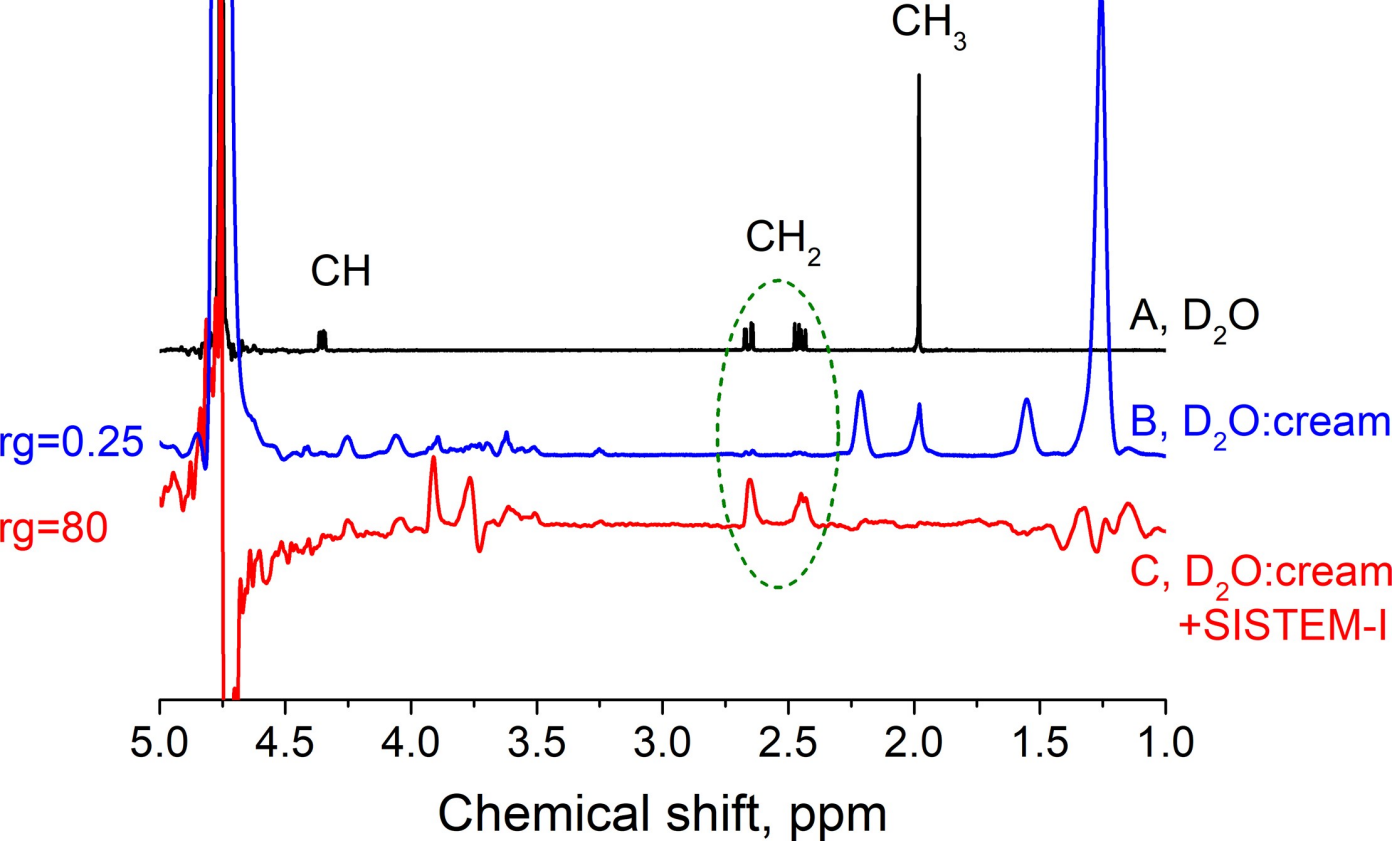
^1^H NMR spectra of NAA in D_2_O (MS1) and homogeneous field (A), NAA in aqueous-cream solution (MS2) with poor field homogeneity (B) and same solution acquired by SISTEM-I (C). Water (4.7 ppm) and lipid resonances (0.9–2.2 ppm range) [57] dominated the routine NMR spectrum (B) were effectively suppressed by SISTEM-I (C), allowing to increase the receiver gain 320 fold. The NAA-CH_2_ resonance was well prepared by SISTEM-I (dashed oval). Interestingly, two other resonances at ≈ 3.8 ppm were also prepared by SISTEM-I. These chemical shifts coincide well with those of D-lactose β (CH_2_: 3.96 ppm and 3.81 ppm) [56]. Parameters of SISTEM-I: *τ*_1_ = 8 ms, *τ*_*d*_ = 1 s, number of scans, NS, was 8, no phase cycling, WALTZ-16 decoupling with 2.5 kHz RF- amplitude. ZQCs were suppressed with CHIRP pulses accompanied by gradients [39, 47]. Spectra (A) and (B) were acquired with NS = 1. Average SNR of NAA-CH_2_ signals was 23 (B) and 50 (C) for the given parameters and exponential line broadening of 5 Hz.

**Fig 4:**
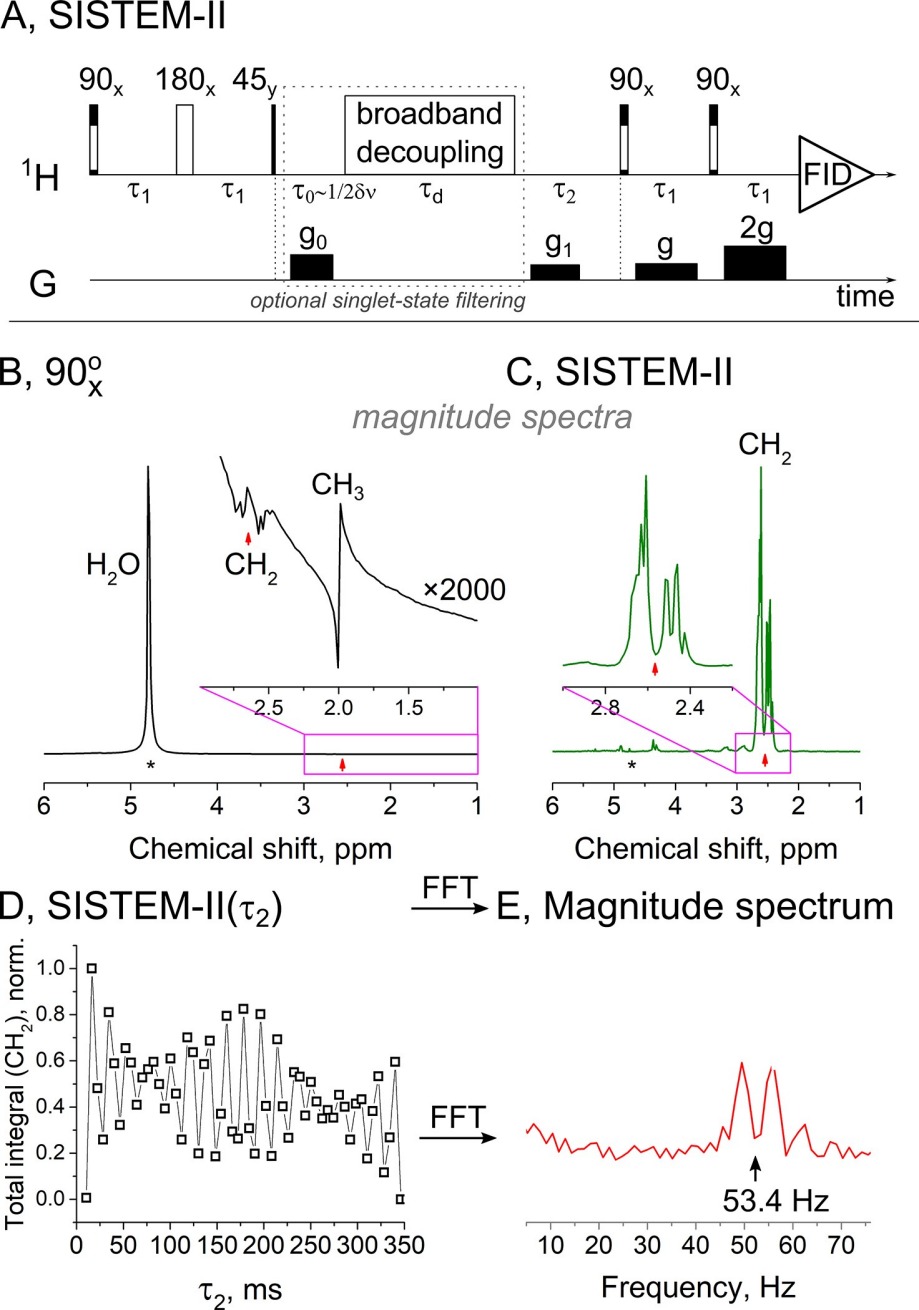
Performance of SISTEM-II on a 7 T MRI and measurement of chemical shift difference and J-coupling constants in inhomogeneous field of MRI. Schematic view of SISTEM-II (A). Optional in this case broadband decoupling (part in the dashed box) was omitted in experiments to decrease SAR (*τ*_0_, *τ*_*d*_ = 0). In comparison to a pulse-acquisition magnitude spectrum (B), the water signal of **MS3** was strongly suppressed by SISTEM-II, while the NAA-CH_2_ signal was maintained (C, magnitude, *τ*_1_ = 12 *ms*, *τ*_2_ = 8.2 *ms*). Note that the spectrum has a complex shape, that does not allow to determine $\Delta \delta_{CH_{2}}$ directly. Instead, the chemical shift difference $\Delta \delta_{CH_{2}}$ was encoded into the signal by variation of *τ*_2_, and determined by Fourier transforming the summed amplitudes of the SISTEM-II signal acquired for different *τ*_2_ (D). A well-resolved doublet at $\Delta \delta_{CH_{2}}=52.7\pm 0.6$ Hz or ≈0.176±0.002 ppm with the splitting of $\Delta J=J_{CH_{2}^{a}}^{CH}-J_{CH_{2}^{b}}^{CH}\approx 6.2\pm 0.6$ Hz was obtained (E) despite a poor field homogeneity (worse than 30 Hz linewidth). Inserts show NAA-CH_2_ resonances (B, C). Small red arrows indicate the center of NAA-CH_2_ resonances. SISTEM-II experimental time is 5 s for a single scan (C); 55 points (D) were acquired in 4.5 minutes. NAA/background signal in (B) is ≈10^−4^ and ≈10 in (C). Average SNR of NAA-CH_2_ signals was 300 (B) and 1300 (C) for the given experimental parameters.

Step 1: In the first stage, thermal spin magnetization is transferred to the population of the singlet state and zero-quantum coherences (ZQCs). To reach this goal, several methods were proposed [43–46]. We chose the method proposed by Sarkar et al. [39] (Sarkar-II) because it is independent of frequency offsets and uses only hard RF-pulses and gradients. To suppress high order quantum coherences, we added the following spoiler gradient (**Figs 2A and 4A**). To suppress ZQCs one can also add a Thrippleton—Keeler filter too (used in **Fig 3** and **S2 Fig in S1 File**) [39, 47].

Step 2: The second stage is used to encode a dedicated feature (e.g. chemical exchange [39], diffusion [48] or pH). Here, we encode the chemical shift difference of NAA-CH_2_ protons by introducing a free evolution interval *τ*_2_ (**Figs 2A** and **4A**). During this stage, the in-phase, ${\hat{ZQ}}_{x}={\hat{I}}_{x}{\hat{S}}_{x}+{\hat{I}}_{y}{\hat{S}}_{y}$ and out-of-phase, ${\hat{ZQ}}_{y}={\hat{I}}_{y}{\hat{S}}_{x}-{\hat{I}}_{x}{\hat{S}}_{y}$, ZQCs mutually alternate [39, 49]:

$${\hat{ZQ}}_{x}\overset{2\pi \tau (\nu_{I}{\hat{I}}_{z}+\nu_{S}{\hat{S}}_{z})}{\to }{{\hat{ZQ}}_{x}\;\cos\left(2\pi \delta \nu \tau \right)+\hat{ZQ}}_{y}\;\sin\left(2\pi \delta \nu \tau \right)$$ (1)

where *ν*_*I*_ and *ν*_*S*_ are Larmour precession frequencies of two spins I and S and *δν* = *ν*_I_−*ν*_S_ is their chemical shift difference.

Step 3: The goal of the third stage is to suppress the signals from unwanted spins and spin states. We implemented two variants: strong broad-band decoupling on the NMR device (**SISTEM-I, Figs 2A and 3, S2 Fig in S1 File**), and Only-Parahydrogen SpectroscopY with double quantum coherence filter (OPSYd) on the MRI system (**SISTEM-II, Fig 4**) [42, 50]. Note that the deposition of RF energy by OPSYd is much lower than that of decoupling and water/fat saturation. Optional broad-band decoupling, however, not only suppresses background signal but also sustains long-lived singlet spin states. This feature of LLSs offers an additional MRI contrast [32]. An alternative background suppression technique is a specialized singlet spin order filter (T_00_-filters) [51, 52].

Step 4: During the fourth stage, LLSs and ZQCs are transferred back to observable magnetization. For SISTEM-I, we used the second block of the Sarkar-II sequence ("out of phase echo" block, **Fig 2A**). For SISTEM-II, this function is accomplished with OPSYd (**Fig 4A**).

Step 5: During the final stage, MR signal is acquired e.g. by pulse-acquisition experiments or, possibly, imaging.

## 3. Results

### 3.1. Properties of the filter

The performance of SISTEM-I (**Fig 2A**) (known as “Sarkar-II”) is described in Ref. [39] and (**SM**). Again, this pulse sequence was chosen without modifications from Ref. [39] and it seems to be the most simplest, transmitting frequency independent, broadband singlet spin order selecting pulse sequence. The interval *τ*_1_ selects systems with the desired J-coupling constant, *J*_*AB*_, and the interval *τ*_2_ selects the system with the desired chemical shift difference, *δv*, as the maximum magnetization to singlet order transfer and back occurs for $\tau_{1}=\frac{1}{4|J|}$ and $\tau_{2}=\frac{1}{\delta \nu }$ (**Fig 2B and 2C**).

In the first part of SISTEM-I (“M2S”), for a weakly coupled two spin system, the Boltzmann polarization or magnetization is transferred to a singlet-triplet population imbalance, $Q_{SO}=\langle {\hat{Q}}_{SO}\rangle =\langle -\frac{4}{3}(\hat{S}∙\hat{I})\rangle$ [53], according to the following, simple equation:

$$Q_{S0}=\frac{1}{4}\sin\left(2\pi J\tau_{1}\right)[1-\cos\left(2\pi \delta \nu \tau_{2}\right)].$$ (2)

However, already for 2-spin systems in the intermediate coupling regime, the matter becomes more complicated, and the polarization transfer behavior deviates from this simple equation (**Fig 2B, 2C and 2F**). We exemplified the matter with two spin systems with $\theta =\frac{1}{2}Arctan\left(|\frac{J}{\delta \nu }|\right)≅9^{o}$ (AB type) and θ≅1^o^ (AX type). The angle *θ* tends to zero, *θ*→0, when the system is weakly coupled (AX type), on the other hand, when *θ*→45^*o*^ the system is considered to be strongly coupled (AB or even A_2_ type). A θ≅9° corresponds to two NAA-CH_2_ protons at pH 5 (**S1 Table in S1 File**).

DeVience et al [44] demonstrated that the intervals *τ*_1_ and *τ*_2_ can be chosen such that almost perfect suppression of NAA is achieved, while signals of aspartate are preserved. Here, we found other feature of this filter: periodic filtration (**Fig 2D, 2E and 2G**).

First, we optimized the parameters of SISTEM-I for the maximum retained signal of NAA (**Fig 2B and 2C**) after signal filtration. Then we applied this pulse sequence to other two-spin systems with various chemical shift differences and J-coupling constants (**Fig 2D and 2E**). As one can see, and it follows from Eq 3, the pulse sequence is insensitive to the sign of J-coupling constant (the sign of the signal is irrelevant in this case), and systems with odd multiples of chemical shift difference pass through the filter. The criteria for the AX systems, that pass through the SISTEM-I filter, follow from Eq 2 and **Fig 2**:

$$J=\left(2i+1\right)J_{0},\;\mathrm{with}\;i\in \mathbb{Z},\;\mathrm{and}\;\tau_{1}=\frac{1}{4\left|J_{0}\right|}$$ (3)

$$\left|\delta \nu \right|=\left(2n+1\right)|\delta \nu_{0}|,\;\mathrm{with}\;n\in \mathbb{N},\;\mathrm{and}\;\tau_{2}=\frac{1}{2\delta \nu_{0}}$$ (4)

Thus, the pulse sequence not only suppresses uncoupled spins like H_2_O, but also two spin systems that do not satisfy these criteria (Eqs 3 and 4). Put differently, the SISTEM-I filter is not specific to a single set of *J* and *δν*, but to multiples of it as well. The calculation of SISTEM-I performance for the 3 spin system of NAA is given in Supporting Materials.

Free evolution intervals *τ*_2_ (**Fig 2A**) can be substituted by broad-band CHIRP pulses accompanied by gradients, i.e. by Thrippleton–Keeler filter that suppresses ZQCs [47]. In this case, the pulse sequence is insensitive to the chemical shift difference [39]. We used this approach in our high-resolution NMR experiments (**Fig 3, S2 Fig in S1 File**).

### 3.2. Implementation of SISTEM-I on a high-resolution NMR spectrometer

It should be noted that SISTEM-I was designed with a 2-spin-½ system in mind [39], however, it can also be applied to three- and multi-spin systems [54]. NAA is effectively a 3 spin-½ system (CH-CH_2_, **S1 Scheme**), therefore the efficiency of the sequence was reduced. For NAA at pH 5, the maximum retained thermal magnetization after SISTEM-I is predicted to be 0.45 (**S1 Fig in S1 File**), while for the two-spin system it is 2/3 (**Fig 2**). An optimal *τ*_1_≈ 12 ms was found for NAA-CH_2_ protons and SISTEM-I sequence (**S2 Fig in S1 File**).

Still, the LLS was successfully populated, and the lifetime of T_LLS_ ≈ 6.5 s was almost 6 times longer than T_1_ ≈ 1 s (**S2 Fig in S1 File,** at 14.1 T with 2.5 kHz WALTZ-16 decoupling [55]). However, note, we do not use the long live-time hereafter as a contrast.

### 3.3. Suppression of water and fat signals with SISTEM-I on a high-resolution NMR spectrometer

Next, we used a 1:1 mixture of water and dairy cream with 30% fat content (**MS2**, **Fig 3B and 3C**). To evaluate the performance of the method in a field with poor homogeneity, we refrained from shimming. The resulting linewidth was irregular with a full width at half maximum of ≈ 30 Hz, resembling in vivo conditions.

As expected, the spectrum of a standard, 90° pulse-acquisition experiment (without any suppression techniques) was dominated by fat and water resonances, while only very little NAA was apparent (**Fig 3B**). Using SISTEM-I, however, the fat and water signals were strongly suppressed, allowing to increase the receiver gain (rg) 320 times (linear, **Fig 3C**).

The SISTEM-I spectrum showed well-resolved resonances of NAA-CH_2_, but no signal from the NAA-CH_3_ group, which normally dominates the NAA spectrum. Besides, some water signal and some residues of lipid-(CH_2_)_n_ were found at ≈ 1.3 ppm. The signals in the range 3.5–4.5 ppm were tentatively attributed to lactose; two remained peaks at 3.9 and 3.76 ppm were attributed to CH_2_ protons of D-lactose β (3.96 ppm and 3.81 ppm) [56]. Because of similar structure and similar NMR-parameters this pair of spins could pass through the SISTEM-I filter.

### 3.4. SISTEM-II on a 7 T small animal MRI system

For in vivo applications, care must be taken when using decoupling because much energy may be deposited into the tissue. To circumvent this issue, we propose to use OPSYd filter instead of decoupling (SISTEM-II, **Fig 4A**). Note that in this case, an additional magnetization to singlet transfer stage is no longer required: OPSYd effect on zero-quantum coherences and LLS is described in **SM**.

We implemented SISTEM-II on a preclinical 7 T MRI. Again, the spectrum of a standard pulse-acquisition method was dominated by water signal three orders of magnitude larger than that of NAA (**MS3**). Water and NAA-CH_3_ signals were strongly suppressed by SISTEM-II, while NAA-CH_2_ signals were retained (**Fig 4B** and **4C**). Note, that the shape of the resonance is complex and not straight forward to interpret, however, the chemical shift difference, $\Delta \delta_{CH_{2}}$, still can be measured indirectly.

We used SISTEM-II to encode the chemical shift difference of NAA-CH_2_ protons ($\Delta \delta_{CH_{2}}$) in the magnitude of the SISTEM-II signal as a function of *τ*_2_; other unwanted resonances were suppressed (**Fig 4**).

To achieve this, we took advantage of the fact that the evolution of the ZQCs during the encoding phase of SISTEM-II depend on $\Delta \delta_{CH_{2}}$ (Eqs 1 and 2). Thus, by variation of *τ*_2_ and Fourier transform of the resulting signal, we found a doublet centred at $\Delta \delta_{CH_{2}}=52.7\pm 0.6$ Hz (at 7 T or 0.176 ppm). The splitting was caused by an additional modulation of the SISTEM-II signal induced by spin-spin coupling with a third nucleus, the NAA-CH proton (**Fig 4E**). This splitting equals to 6.2±0.6 *Hz* and corresponds to $\Delta J=J_{CH_{2}^{a}}^{CH}-J_{CH_{2}^{b}}^{CH}$ (**S1 Table in S1 File**) [58, 59]. Note that it is impossible to determine these parameters with this precision by MRI in a simple pulse-acquisition experiment with a common in MRI magnetic field homogeneity worse than 30 Hz.

Note, that here we did not apply an optional in this case broadband decoupling (**Fig 4A**) to preserve a singlet spin state as it was done in SISTEM-I, as a result, the singlet spin state is only transient state in the interval *τ*_2_. The broadband decoupling (singlet state filtering) could improve background suppression, albeit by reducing the NMR signal and increasing specific absorption (SAR).

### 3.5. pH measurement with SISTEM-II on a 7 T small animal MRI system

**Fig 5:**
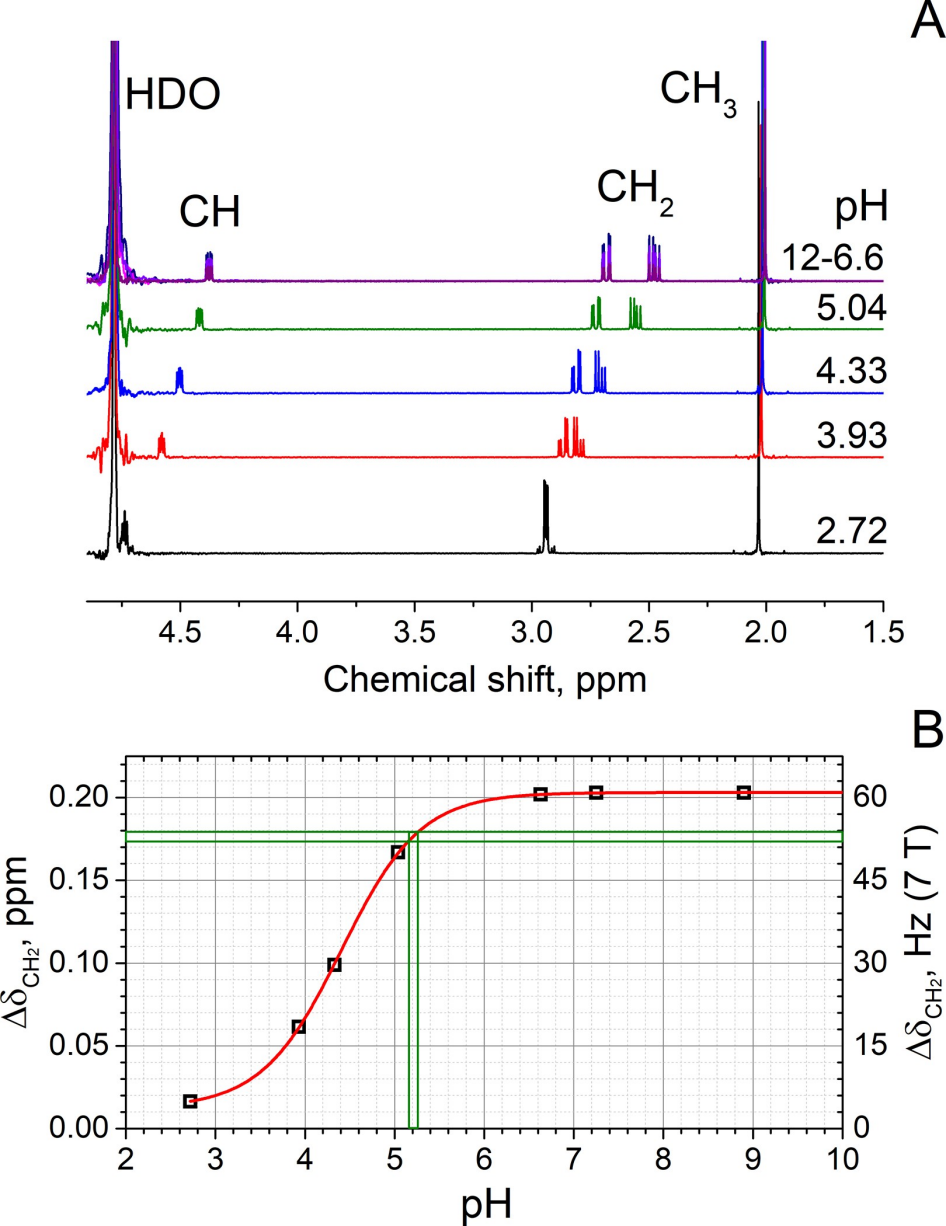
NAA NMR spectra and $\Delta \delta_{CH_{2}}$ of NAA-CH2 as a function of pH. Seven high resolution, ^1^H NMR spectra (A) of **MS1** with different pH were acquired to determine the effect of pH on $\Delta \delta_{CH_{2}}$ (B). Henderson-Hasselbalch equation in a form: $\Delta \delta_{obs}=\Delta \delta_{p}+\frac{\Delta \delta }{1+10^{pKa-pH}}$ [63] with Δ*δ*_*p*_ = 0.013±0.0006 ppm, Δ*δ* = 0.19±0.0006 ppm, pKa = 4.407±0.006 (B, line) was fitted to the data (B, squares). The $\Delta \delta_{CH_{2}}=52.7\pm 0.6\;Hz$ determined by SITEMS-II (**Fig 4E**) corresponded to a pH of 5.21±0.05 (cross-section of two green boxes), which corresponded well to the value measured by electrode 5.15. Fitted chemical shifts and J-coupling constants as a function of pH are listed given in **SM** together with fitting parameters.

In general, selectively preparing specific resonances and suppressing background often opens the door for new applications. Now that the chemical shift difference of NAA-CH_2_ can be measured much more precisely than the poor homogeneity of the magnetic field would normally allow, we can use this information to assign the pH of the sample. For this purpose, we acquired seven high-resolution NMR reference spectra of NAA at pH 3–10. $\Delta \delta_{CH_{2}}$ was found to collapse for low pH approaching 2, and to reach a maximum for a pH of 7 or more (≈ 0.2 ppm, **Fig 5**). According to these data, the $\Delta \delta_{CH_{2}}=52.7\pm 0.6\;Hz$ measured in the 7 T MRI corresponds to a pH of 5.21±0.05. This finding compares well with the pH value of 5.15 measured with an electrode.

The method does not require any external reference, nor is any correction of magnetic field homogeneity or susceptibility needed, because the chemical shift difference, $\Delta \delta_{CH_{2}}$, within one and the same molecule is used as a pH meter [60, 61]. Unfortunately, though, for NAA, most of the $\Delta \delta_{CH_{2}}$ variation is just below the most interesting physiological range of pH. Still, other molecules with more appropriate properties may be identified. Note, that the effect of ionic strength and temperature on correlation of pH value with NMR parameters for the chosen target molecules has to be also considered [62].

## 4. Discussion

Among the multitude of applications suggested for LLS, we showed here that LLS can be used to selectively prepare the signals of a metabolite, NAA, in the presence of water, inhomogeneous magnetic fields and highly concentrated fatty solutions. This facilitates quantification because the background is greatly suppressed.

One application of this technique may be the measurement of endogenous brain metabolites, either of a whole-brain at once [7] or as a part of localized spectroscopy or imaging methods. Another application may be imaging of biodistribution of drugs. An example would be ethosuximide (ETX, Zarontin) [64], a medication used to treat absence seizures. ETX has an isolated CH_2_ group of the two weakly coupled protons, which is ideal for SISTEM-I (**Fig 2**). In this case, up to 2/3 of the thermal Boltzmann signal could be observed. Here, the utility of the method was demonstrated on a three spin-½ system of NAA with two CH_2_ protons in the intermediate coupling regime.

SISTEM is not limited to CH_2_ groups only, one can exploit other fragments such as CH-CH_3_ or other coupled spins [41]. The spin system of lactate (abundant metabolite) e.g., comprises a weakly coupled CH-CH_3_ group of spins with J-coupling constant of 7 Hz; in this case, up to 1/3 of the Boltzmann signal can be observed with SISTEM-I.

Nowadays, numerous pulse sequences exist, including recently proposed broad-band generalized magnetization-to-singlet-order transfer [65]. The optimum performance of such sequences covers a significant range of coupled spin systems. The molecule of choice, available equipment and SAR will dictate the use of the filtering method.

It is worth to mention that although double quantum and zero quantum (DQ/ZQ) filters are used in MRS [66] the SISTEM approach is quite different. LLS filtering techniques select not only multi-spin systems but the systems with the specific values of chemical shift and J-coupling constant (**Fig 2**).

Besides imaging the distribution and measuring the concentration of molecules, SISTEM may be used to improve the ability of NMR to probe various tissue properties such as pH. The approach presented here is particularly advantageous because it is independent of the magnetic field homogeneity and requires no external reference. It was already suggested to use heteronuclear (^31^P, ^13^C, ^15^N, e.g.) J-coupling constants and chemical shifts to determine pH value by MRI [61, 62, 67]. X-nuclei methods benefit from little to no background, while ^1^H methods offer a high detection sensitivity and do not require X-nuclear capabilities.

While the conditions investigated here were used to approach the situation in vivo, obviously, only in vitro experiments were presented here. If the method has any value for in vivo biomedical applications has yet to be shown. Still, these first results are promising. We are currently trying to bring together this spectral editing technique with spatial encoding MRI methods (PRESS, CSI, e.g.) to approach with this method in vivo and possibly clinical applications and to assess the gained SNR.

## 5. Conclusion

We showed that SISTEM can be used to selectively prepare and measure the signals of chosen metabolites or drugs in the presence of water, highly concentrated fatty solutions in homogenous and inhomogeneous fields. Very strong suppression of unwanted signals was observed on an NMR and MRI system with low RF-power deposition (no saturation pulses were used in MRI) that is beneficial for in vivo MRI. The chemical shift difference of the NAA-CH_2_ protons was encoded into the SISTEM signal and used for measuring pH. The sequences greatly suppress all isolated/uncoupled protons (e.g. some CH_3_ groups, water), however, some multi-spin systems can still pass the filter. In vivo tests are required to see if this method has a biomedical relevance.

## Supporting information

S1 File NMR spectra of all substrates listed in the methods and chemical shifts and J-coupling values for NAA as a function of pH. (PDF)

S1 Data (ZIP)

S1 Scheme Chemical structure of N-acetyl-l-aspartic acid (NAA). The methyl protons (a, b) were used for SISTEM. The protons have J-coupling constant of 16–17 Hz with a chemical shift of 2.5–3 ppm depending on pH (**SM**).
(TIF)

## Abbreviations

CW: continuous-wave excitation
LLS: long-lived spin states
MRS: Magnetic resonance spectroscopy
MS: Model Solution
NAA: N-acetyl-L-aspartic acid
OPSYd: Only-Parahydrogen SpectroscopY with double quantum coherence filter
SAR: specific absorption rate
SISTEM: singlet-state encoded magnetic resonance spectroscopy
SM: supporting materials
SUCCESS: suppression of undesired chemicals using contrast-enhancing singlet states
