# Supplementary material for: In vitro singlet state and zero-quantum encoded magnetic resonance spectroscopy: Illustration with N-acetyl-aspartate
Source: PLoS One. 2020 Oct 1;15(10):e0239982. doi: 10.1371/journal.pone.0239982 (PMC7529218; doi:10.1371/journal.pone.0239982)
Supplement: S1 File — (PDF) [file pone.0239982.s001.pdf]

Supporting materials

In vitro singlet state and zero-quantum encoded magnetic resonance spectroscopy: illustration with N-acetyl-aspartate

Andrey N. Pravdivtsev,*[a] Frank D. Sönnichsen[b] and Jan-Bernd Hövener[a]

[a] Section Biomedical Imaging, Molecular Imaging North Competence Center (MOIN CC), Department of Radiology and Neuroradiology, University Medical Center Kiel, Kiel University (http://www.moincc.de/)
Am Botanischen Garten 14, 24118, Kiel, Germany
E-mails: andrey.pravdivtsev@rad.uni-kiel.de; jan.hoevener@rad.uni-kiel.de

[b] Otto Diels Institute for Organic Chemistry, Kiel University, Otto Hahn Platz 5, 24098, Kiel, Germany

Table of content

1. N-acetyl-L-aspartic acid parameters S-3
2. Sarkar-II: magnetization to zero-quantum coherences and singlet spin state S-4
3. OPSYd: filtering capabilities S-5
4. SISTEM-I applied to NAA: simulations S-6
5. SISTEM-I optimization for NAA S-7
6. DL-Lactic acid S-8
7. L-Alanine S-9
8. Creatine monohydrate S-10
9. Choline chloride S-11
10. L-Glutamic acid S-12
11. Myo-Inositol S-13
12. References: S-14

1. N-acetyl-L-aspartic acid parameters

Table S1. NMR parameters of NAA. Chemical shifts, δ, (in ppm) and J-coupling constants, J, (in Hz) of NAA protons as a function of pH (Figure 5). NMR parameters are constant at pH above ~6. NMR spectra acquired on a Bruker Avance II 600 MHz were analyzed. The pH values are the electrode values read on a pH meter that was calibrated using protic buffer solutions. All the samples were prepared in D_2O.

pH reading	$\delta(CH_3)$	$\delta(CH_2^a)$	$\delta(CH_2^b)$	$\delta(CH)$	$\delta(CH_2^b) - \delta(CH_2^a)$	$J(CH_2)$	$J(CH_2^a - CH)$	$J(CH_2^a - CH)$
2.72	2.03306	2.93212	2.9485	4.73791	0.01638	-17.2	6	6
3.93	2.02189	2.80271	2.86423	4.58047	0.06152	-16.6	7.2	4.8
4.33	2.01658	2.71001	2.80899	4.504	0.09898	-16.2	7.9	4.5
5.04	2.00821	2.55875	2.72563	4.4191	0.16688	-15.9	9.5	3.9
6.63	2.0041	2.48064	2.68244	4.37907	0.2018	-15.7	10.2	3.7
12	2.00422	2.47755	2.68048	4.37728	0.20293	-15.7	10.2	3.7

The following Henderson-Hasselbalch equation was used to fit the chemical shift as a function of pH (**Figure 5B**):

$$\Delta\delta_{obs} = \Delta\delta_p + \frac{\Delta\delta}{1 + 10^{pKa-pH}}$$

Where $\Delta\delta_{obs} = \delta(CH_2^b) - \delta(CH_2^a)$ (Table S1) is the observed chemical shift difference, $\Delta\delta_p = 0.013 \pm 0.0006$ ppm is the chemical shift of the fully protonated form, $\Delta\delta = 0.19 \pm 0.0006$ ppm is the chemical shift difference change associated with the pH variation, and pKa=4.407±0.006. These parameters, $\Delta\delta_p$ $\Delta\delta$, n, and pKa are the result of fitting this equation to the $\Delta\delta_{obs}$(pH) data.

Table S2. T_1 relaxation times of NAA in D_2O at pH 6.

	CH_3	CH_2^a	CH_2^b	CH
T_1, s	1.77	0.88	0.79	4.2

2. Sarkar-II: magnetization to zero-quantum coherences and singlet spin state

Magnetization to singlet spin state is realized in Sarkar-II (SISTEM-I) sequence with the following RF-pulses and intervals:

$$\left(\frac{\pi}{2}\right)_x - \tau_1 - (\pi)_x - \tau_1 - (\varphi)_y - PFG$$

with $\varphi = \frac{\pi}{4}$, and PFG = pulsed field gradient.

Initial spin state of the two spin-½ system can be represented with $\frac{1}{2}(\hat{I}_z + \hat{S}_z)$ state. The result of the action of this pulse sequence on one spin in a state $\frac{1}{2}\hat{I}_z$ is

$$\frac{1}{2}\hat{I}_z \xrightarrow{\pi/2_x} \frac{1}{2}\hat{I}_y$$

$$\xrightarrow{\tau_1}\xrightarrow{\pi_x}\xrightarrow{\tau_1} -\frac{1}{2}[\hat{I}_y c_J + 2\hat{I}_x\hat{S}_z s_J]$$

$$\xrightarrow{\varphi_y} -\frac{1}{2}\hat{I}_y c_J - (\hat{I}_x c_\varphi - \hat{I}_z s_\varphi)(\hat{S}_z c_\varphi + \hat{S}_x s_\varphi)s_J$$

$$= -\frac{1}{2}\hat{I}_y c_J - \hat{I}_x\hat{S}_z s_J c_\varphi c_\varphi + \frac{1}{2}\hat{I}_z\hat{S}_z s_J s_{2\varphi} - \frac{1}{2}\hat{I}_x\hat{S}_x s_J s_{2\varphi} + \hat{I}_z\hat{S}_x s_J s_\varphi s_\varphi$$

Here $c_\varphi = \cos\varphi$ and $s_\varphi = \sin\varphi$, $s_J = \sin(2\pi J\tau_1)$ and $c_J = \cos(2\pi J\tau_1)$. We are interesting in double spin-state terms therefore will keep $\tau_1 = \frac{1}{4J}$ that gives $s_J = 1$ and $c_J = 0$.

Now we can add the second spin to complete the initial spin state to $\frac{1}{2}(\hat{I}_z + \hat{S}_z)$. This can be done simply by an addition of $I \leftrightarrow$ S permutation in the previous result:

$$\frac{1}{2}(\hat{I}_z + \hat{S}_z) \xrightarrow{\pi/2_x}\xrightarrow{\tau_1=\frac{1}{4J}}\xrightarrow{\pi_x}\xrightarrow{\tau_1=\frac{1}{4J}}\xrightarrow{\varphi_y}$$

$$= -\hat{I}_x\hat{S}_z c_\varphi c_\varphi + \hat{I}_z\hat{S}_x s_\varphi s_\varphi - \hat{I}_z\hat{S}_x c_\varphi c_\varphi + \hat{I}_x\hat{S}_z s_\varphi s_\varphi$$

$$+1\hat{I}_z\hat{S}_z s_{2\varphi}$$

$$-1\hat{I}_x\hat{S}_x s_{2\varphi}$$

$$= -(\hat{I}_x\hat{S}_z + \hat{I}_z\hat{S}_x)c_{2\varphi} + \hat{I}_z\hat{S}_z s_{2\varphi} - \frac{1}{2}\widehat{ZQ}_x s_{2\varphi} - \frac{1}{2}\widehat{DQ}_x s_{2\varphi}$$

Here the elements that are marked "blue" are the result of addition of $\frac{1}{2}\hat{S}_z$ term to the initial spin state, $\hat{I}_x\hat{S}_x = \frac{1}{2}\widehat{ZQ}_x + \frac{1}{2}\widehat{DQ}_x$, with $\widehat{ZQ}_x = \hat{I}_x\hat{S}_x + \hat{I}_y\hat{S}_y$ and $\widehat{DQ}_x = \hat{I}_x\hat{S}_x - \hat{I}_y\hat{S}_y$ being in-phase zero- and double- quantum coherences. Single ($p = \pm 1$), $-(\hat{I}_x\hat{S}_z + \hat{I}_z\hat{S}_x)c_{2\varphi}$, and double ($p = \pm 2$), $-\frac{1}{2}\widehat{DQ}_x s_{2\varphi}$, quantum coherences will be suppressed by the following pulsed field gradients (PFG). Hence, the only remaining terms after "M2S" part of Sarkar-II sequence are:

$$\frac{1}{2}(\hat{I}_z + \hat{S}_z) \xrightarrow{\pi/2_x}\xrightarrow{\tau_1}\xrightarrow{\pi_x}\xrightarrow{\tau_1}\xrightarrow{\varphi_y}\xrightarrow{PFG\,=\,pass(p=0)} \hat{I}_z\hat{S}_z s_{2\varphi} - \frac{1}{2}\widehat{ZQ}_x s_{2\varphi}$$

Hence, the maximum amplitudes of ZQCs and ZZ-order are reached at $\varphi = \frac{\pi}{4} + \pi n$: $\sin(2\varphi) = 1$. The consequent free evolution results in ZQ oscillations (eqs 1 and 2 of the main text) and population of singlet spin state.

3. OPSYd: filtering capabilities

OPSYd filter consists of two 90° RF-pulses and two gradients with $\pm$1:2 ratio:

$$OPSYd: (90) - (\pm G \cdot \tau) - (90) - (2G \cdot \tau) - acquisition$$

$$p: [0] \quad \rightarrow \quad [\pm 2] \quad \rightarrow \quad [-1]$$

After the first 90° RF-pulse, only coherences of the order of $p = \pm 2$ are preserved. This can be achieved if the system is already in a multispin state. The gradients applied before OPSYd suppress all quantum coherences with $p \neq 0$, hence, one can assume that all spin coherencies except of zero-quantum coherences are suppressed before OPSYd. For the two spin-½ systems these states are: $\hat{I}_z\hat{S}_z$, $\widehat{ZQ}_x = \hat{I}_x\hat{S}_x + \hat{I}_y\hat{S}_y$, $\widehat{ZQ}_y = \hat{I}_y\hat{S}_x - \hat{I}_x\hat{S}_y$.

The following terms retain, when the first part of OPSYd is applied to these spin orders:

$$\hat{I}_z\hat{S}_z \xrightarrow{90_x} \hat{I}_y\hat{S}_y = \frac{1}{2}\widehat{ZQ}_x - \frac{1}{2}\widehat{DQ}_x \xrightarrow{DQ-filter} -\frac{1}{2}\widehat{DQ}_x$$

$$\widehat{ZQ}_x \xrightarrow{90_x} \hat{I}_x\hat{S}_x + \hat{I}_z\hat{S}_z = \hat{I}_z\hat{S}_z + \frac{1}{2}\widehat{ZQ}_x + \frac{1}{2}\widehat{DQ}_x \xrightarrow{DQ-filter} +\frac{1}{2}\widehat{DQ}_x$$

$$\widehat{ZQ}_y \xrightarrow{90_x} \hat{I}_z\hat{S}_x - \hat{I}_x\hat{S}_z \xrightarrow{DQ-filter} 0$$

Where $DQ - filter$ means what only states with $p = \pm 2$ pass through and double quantum coherences are $\widehat{DQ}_x = \hat{I}_x\hat{S}_x - \hat{I}_y\hat{S}_y$, $\widehat{DQ}_y = \hat{I}_y\hat{S}_x + \hat{I}_x\hat{S}_y$. The following 90° RF-pulse and gradient convert $\widehat{DQ}_x$ spin order into observable quantum coherences with $p = -1$ (see examples in Ref. (1)).

The first part of SISTEM sequence generates a singlet-triplet imbalance. The operator of singlet-triplet imbalance reads:

$$\hat{Q}_{ST} = -\frac{4}{3}(\hat{I} \cdot \hat{S}) = -\frac{4}{3}(\widehat{ZQ}_x + \hat{I}_z\hat{S}_z)$$

During the free evolution interval before OPSYd, zero-quantum coherences oscillate (eq. 2, main text). Therefore, the state of the system before OPSYd is given by the following spin operators:

$$\hat{Q}_{ST} \xrightarrow{2\pi\tau_2(\nu_I\hat{I}_z + \nu_S\hat{S}_z)} -\frac{4}{3}(\widehat{ZQ}_x \cos(2\pi\delta\nu\tau_2) + \widehat{ZQ}_y \sin(2\pi\delta\nu\tau_2) + \hat{I}_z\hat{S}_z)$$

And consequent 90° RF-pulse together with DQ-filter will retain only the following spin order:

$$-\frac{4}{3}(\widehat{ZQ}_x \cos(2\pi\delta\nu\tau_2) + \widehat{ZQ}_y \sin(2\pi\delta\nu\tau_2) + \hat{I}_z\hat{S}_z) \xrightarrow{90_x} \xrightarrow{DQ-filter} \frac{2}{3}[1 - \cos(2\pi\delta\nu\tau_2)]\widehat{DQ}_x$$

In this way, the OPSYd pulse sequence with a forego interval of free evolution and a coherences suppression gradient allows to pass through only selected zero-order quantum coherences. This filtering technique together with the interconversion of ZQs was used to measure pH in the inhomogeneous magnetic field of MRI (**Figure 5**). $\hat{I}_z\hat{S}_z$ term is time independent; all evolution of the observed signal is going here through the zero-quantum coherences.

4. SISTEM-I applied to NAA: simulations

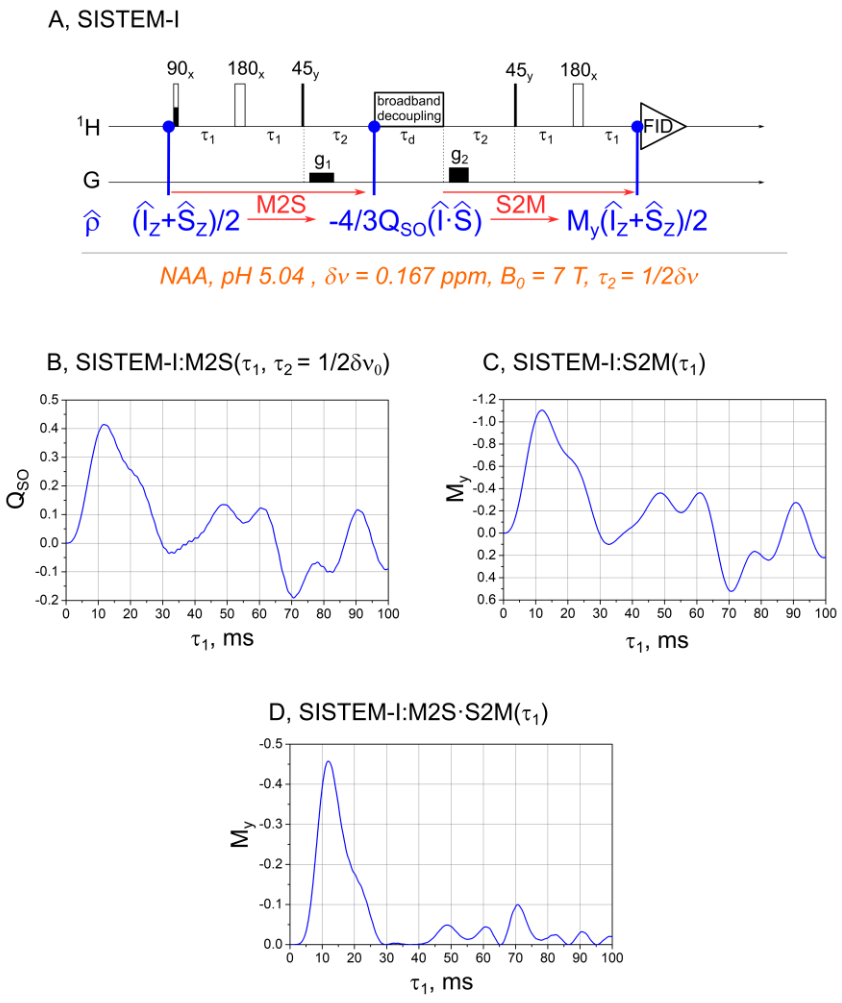

Figure S1. SISTEM-I scheme (A), optimization of SISTEM-I and its elements for a given NAA system at pH 5.04 (Table S1, B-D). Conversion of magnetization to singlet spin state (M2S. B) and back (S2M, C) as a function of interpulse interval τ_1 demonstrates deviation from the predicted $\sin(2\pi\tau_1 J_{AB})$ behavior predicted for a weakly coupled AX spin system(2). For the given system of NAA at pH 5.04, M2S element provide conversion of 100% magnetization, $\frac{1}{2}(\hat{I}_Z + \hat{S}_Z)$, to 40% of singlet-triplet imbalance, $-\frac{4}{3}(\hat{\mathrm{I}} \cdot \hat{\mathrm{S}}) \cdot \mathrm{Q}_{\mathrm{ST}}$, with Q_{ST} = 0.4 , (D); the back conversion demonstrates the same behavior (E), here calculation is started with Q_{ST}=100%. The maximum retained magnetization after SISTEM-I sequence is ≈ 0.45 (D). Interaction with the third CH nucleus results in a quick damping of the polarization transfer.

5. SISTEM-I optimization for NAA

We used an NMR spectrometer to optimize SISTEM-I for preparing the NAA-CH_2 signals. ^{1}H spectra were acquired for 13 different settings of τ_1.

A mono-exponential recovery function was fitted to integrals to extract the T_1-relaxation times (**Figure S2**). To measure the lifetime of LLS, T_{LLS}, SISTEM-I was repeated for various settings of τ_d (**Figure S2**). A two exponential decay function was fitted to the data, and the longer time was attributed to T_{LLS}. The signals were quantified by integrating amplitude spectra.

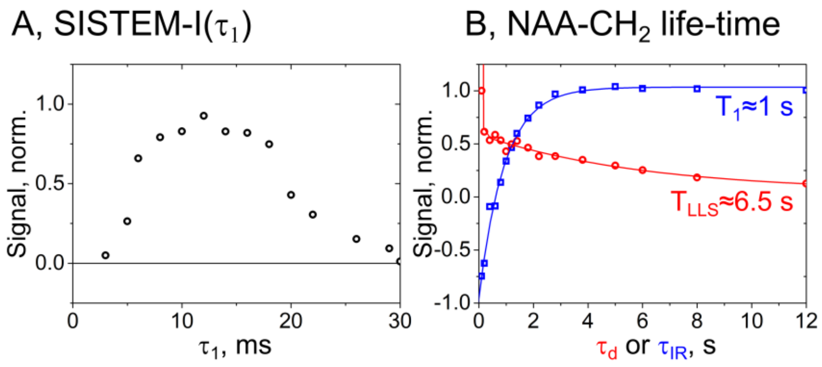

Figure S2. NAA-CH_2 signal acquired by SISTEM-I as a function of τ_1 (A) as well as inversion recovery signals of NAA-CH_2 (B, blue circles) and NAA-CH_2 signal acquired by SISTEM-I as a function of τ_d (B, red squares) with fits (lines). An optimal $\tau_1 \approx 12$ ms (τ_d = 1 s) was found. T_1 of NAA-CH_2 was measured by an inversion recovery (IR) experiment, yielding $T_1 \approx 1$ s (B, blue), there is a lifetime of LLS was measured to $T_{LLS} \approx 6.5$ s (B, red with τ_1 = 8 ms). SISTEM-I parameters: WALTZ-16 decoupling with 2.5 kHz RF-field amplitude at 600 MHz. ZQCs were suppressed with CHIRP pulses accompanied by gradients.(38,48) pH of the sample was 7.25.

6. DL-Lactic acid

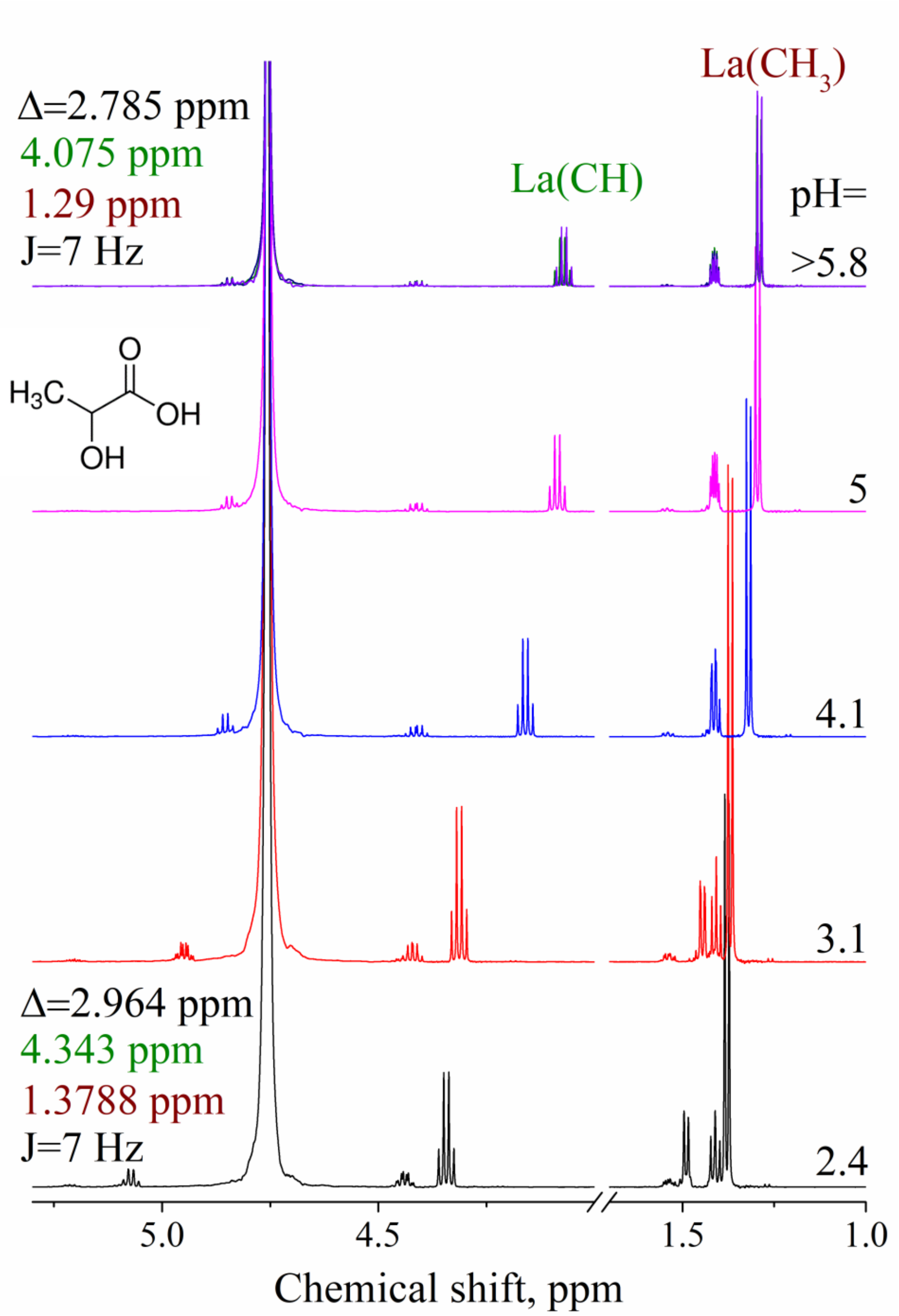

Figure S3. **NMR spectra of 10 mM DL-Lactic acid** (La, Sigma-Aldrich, 69785, CAS: 50-21-5) in D_2O at several pH values. Chemical shifts and J-coupling constants are indicated on figure. NMR spectra were acquired on a Bruker Avance II 600 MHz.

7. L-Alanine

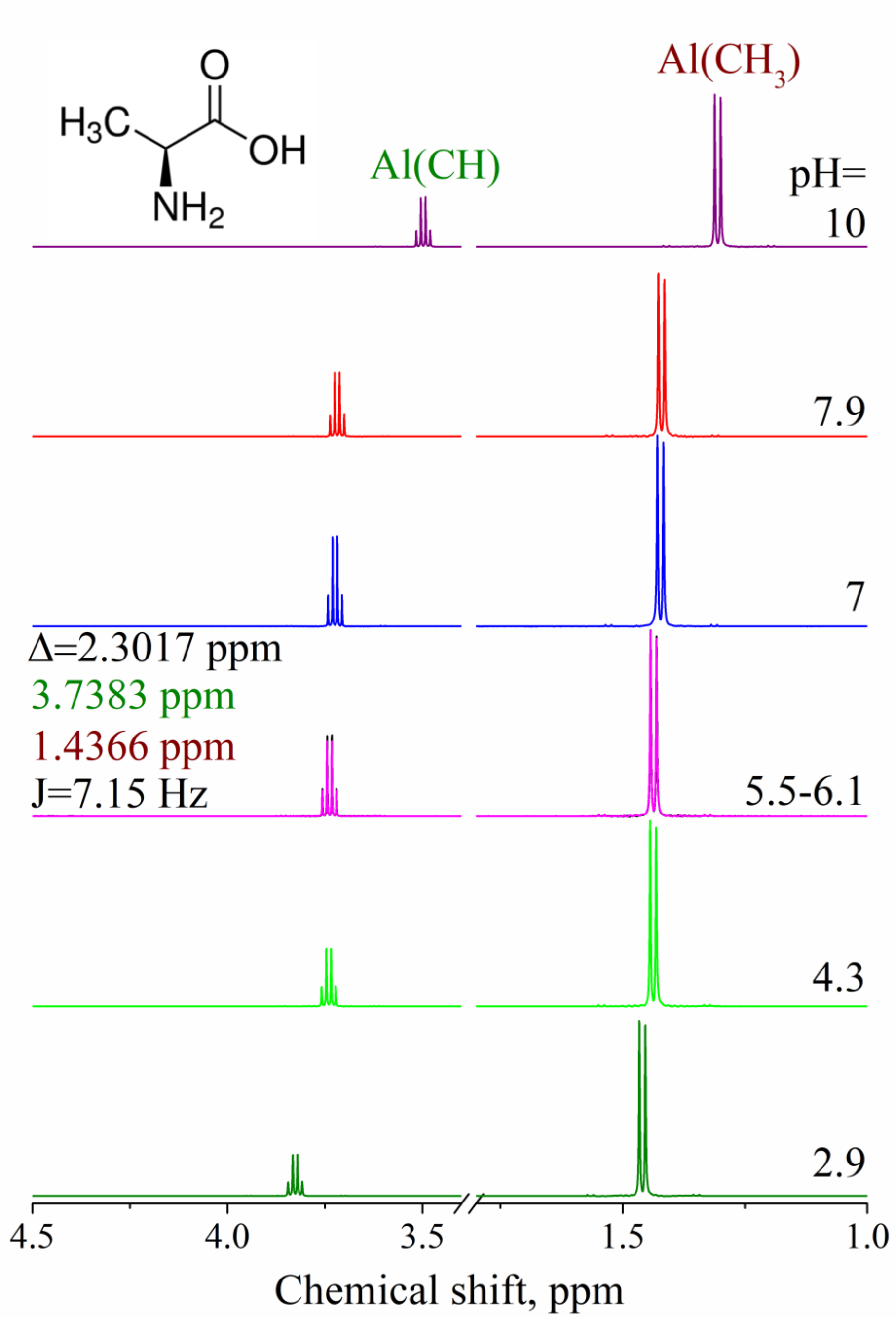

Figure S4. **NMR spectra of 10 mM L-Alanine** (Al, Sigma-Aldrich, A7469, CAS: 56-41-7) in D_2O at several pH values. Chemical shifts and J-coupling constants are indicated on figure. NMR spectra were acquired on a Bruker Avance II 600 MHz.

8. Creatine monohydrate

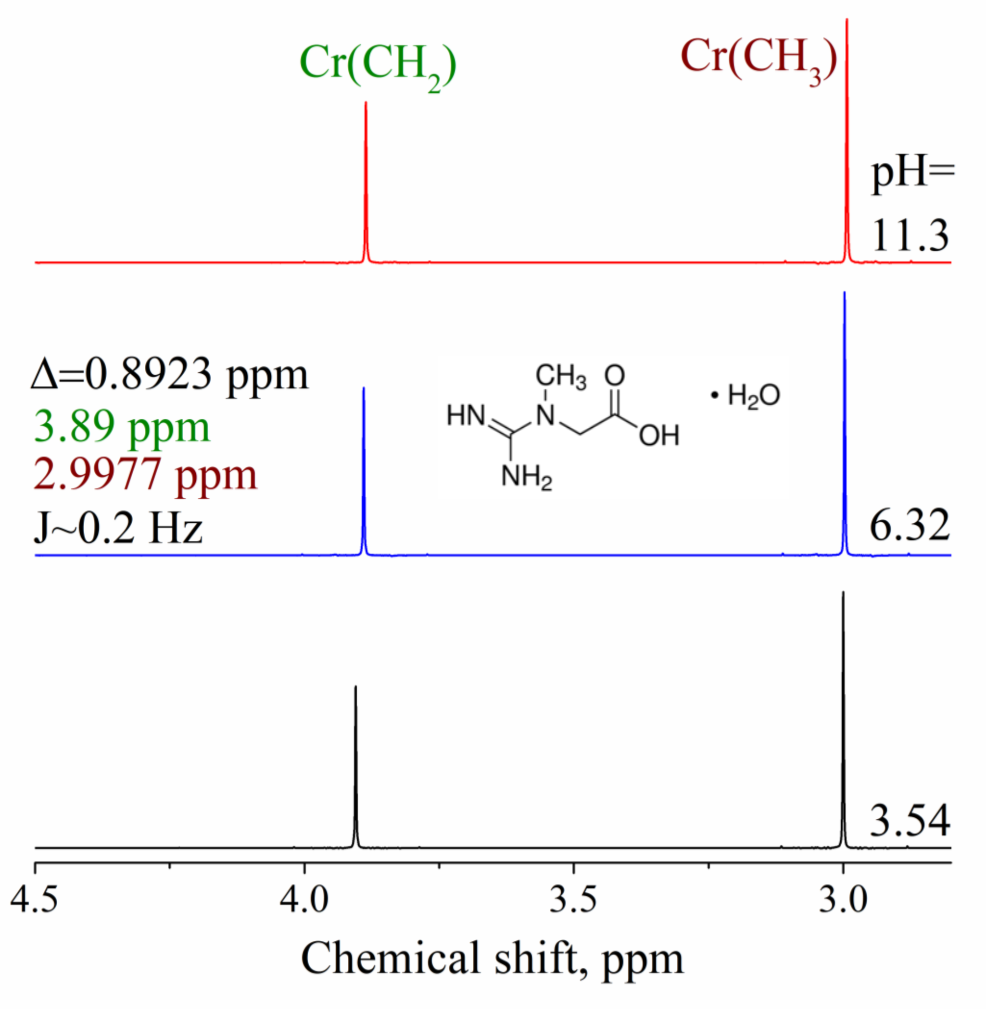

Figure S5. NMR spectra of 10 mM Creatine monohydrate ≥ 98% (Cr, Sigma-Aldrich, C3630, CAS: 6020-87-7) in D_2O at several pH values. Chemical shifts and J-coupling constants are indicated on figure. NMR spectra were acquired on a Bruker Avance II 600 MHz.

9. Choline chloride

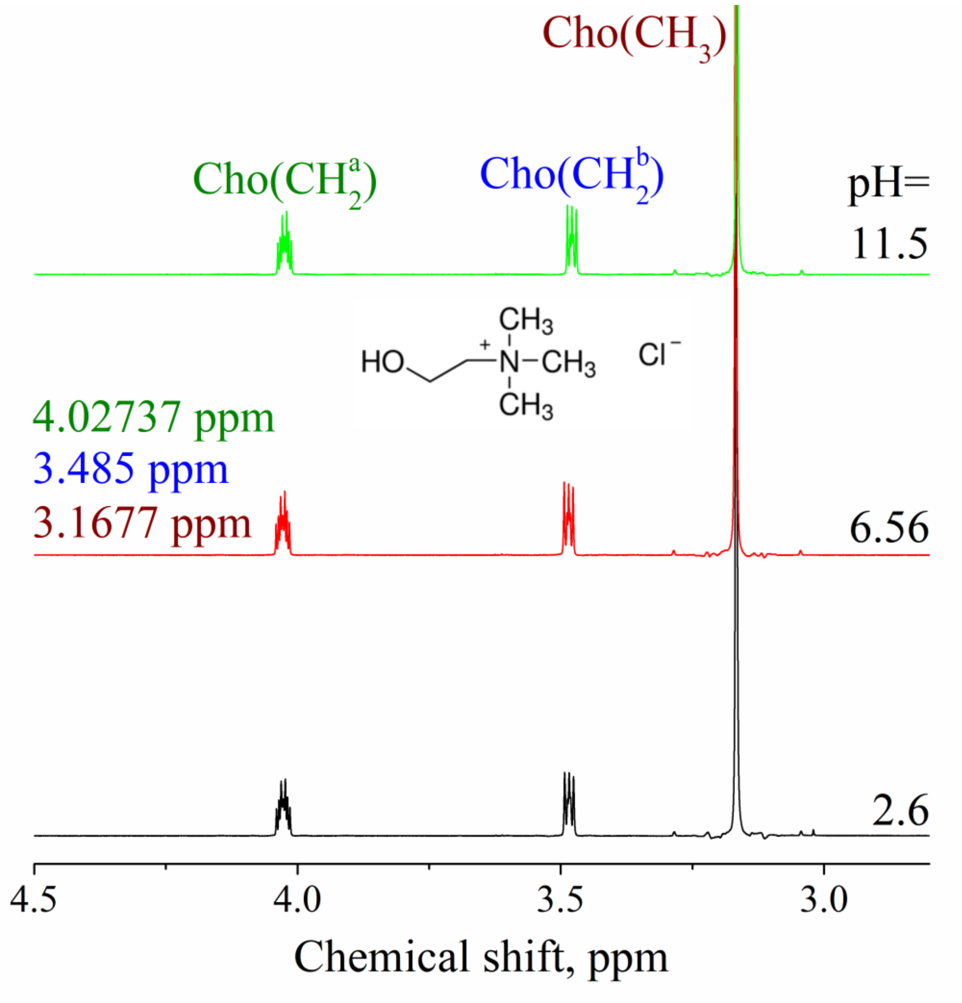

Figure S6. NMR spectra of 10 mM Choline chloride (Cho, Sigma-Aldrich, C7017, CAS: 67-48-1) in D_2O at several pH values. Chemical shifts and J-coupling constants are indicated on figure. NMR spectra were acquired on a Bruker Avance II 600 MHz.

10. L-Glutamic acid

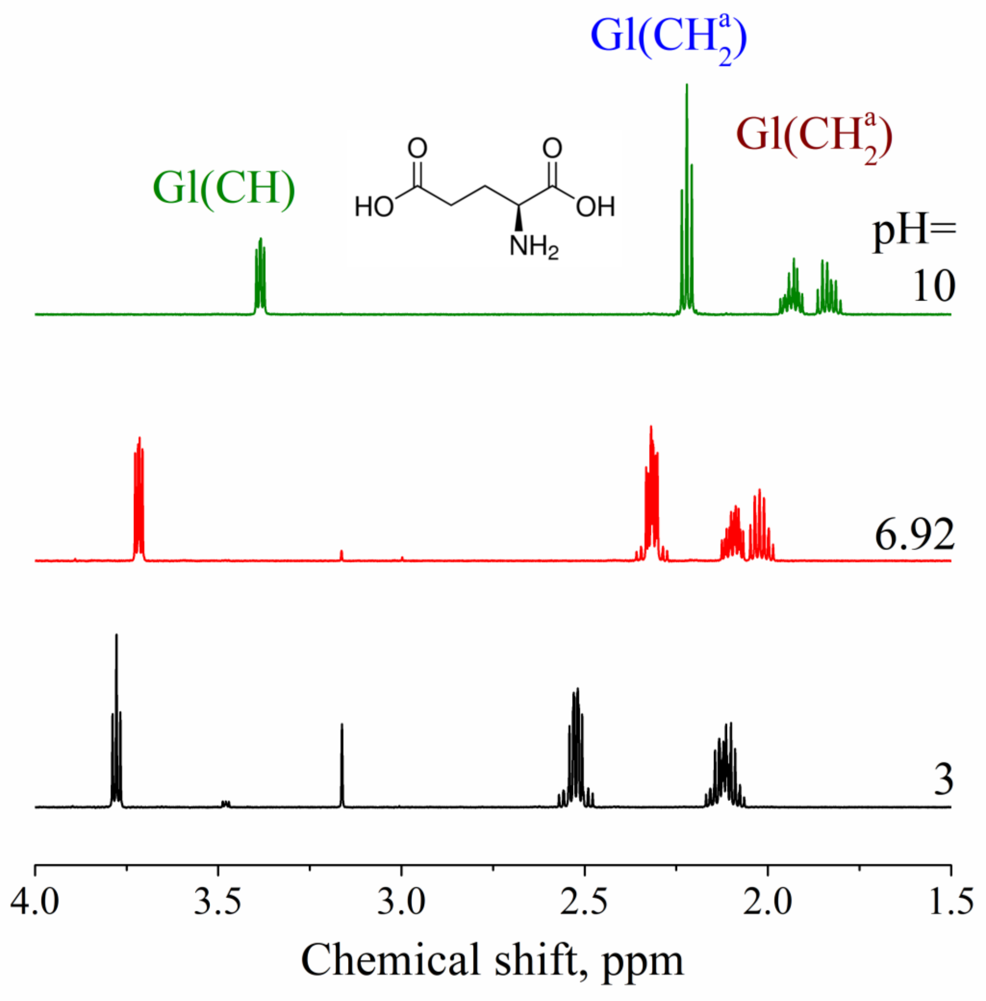

Figure S7. **NMR spectra of 10 mM L-Glutamic acid** (Gl, Sigma-Aldrich, 49449, CAS: 56-86-0) in D_2O at several pH values. NMR spectra were acquired on a Bruker Avance II 600 MHz.

11. Myo-Inositol

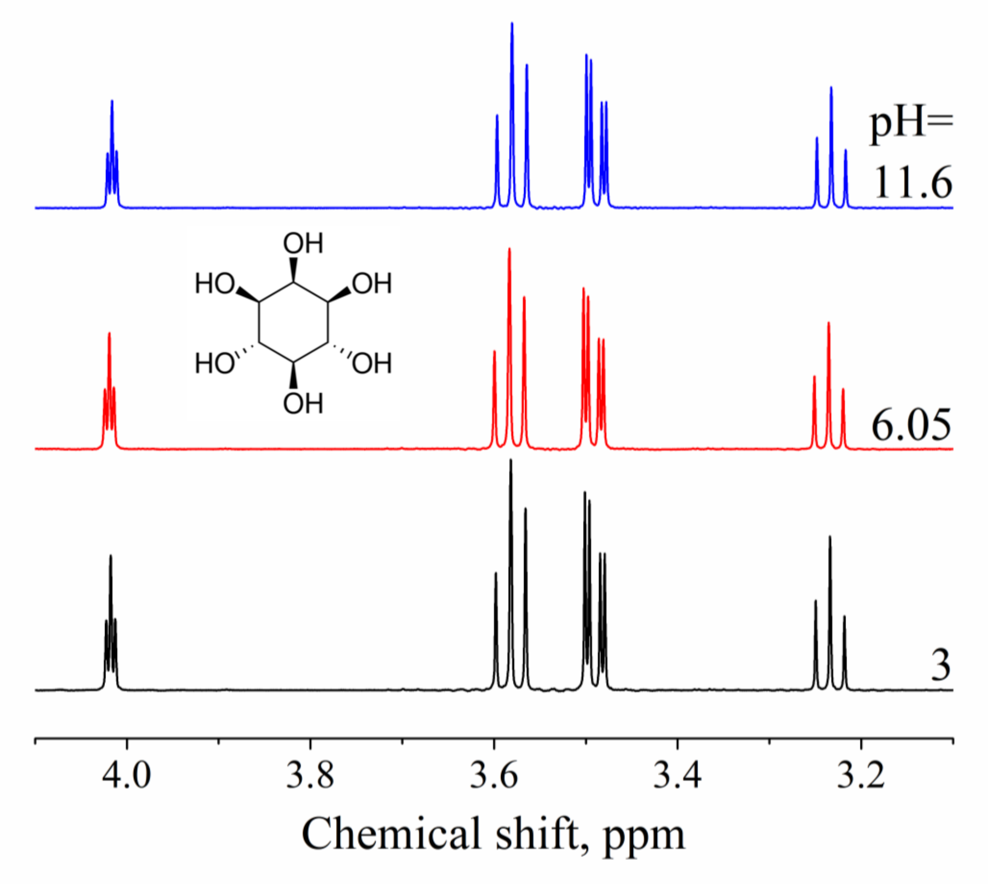

Figure S8. **NMR spectra of 10 mM myo-Inositol** (Sigma-Aldrich, I7508, CAS: 87-89-8) in D_2O at several pH values. NMR spectra were acquired on a Bruker Avance II 600 MHz.

12. References:

1. Pravdivtsev AN, Kozinenko VP, Hövener J-B. Only Para-Hydrogen Spectroscopy (OPSY) Revisited: In-Phase Spectra for Chemical Analysis and Imaging. J Phys Chem A. 2018 Nov 15;122(45):8948–56.

2. Sarkar R, Vasos PR, Bodenhausen G. Singlet-State Exchange NMR Spectroscopy for the Study of Very Slow Dynamic Processes. J Am Chem Soc. 2007 Jan 1;129(2):328–34.
