# Supplementary figures and images for: In vitro singlet state and zero-quantum encoded magnetic resonance spectroscopy: Illustration with N-acetyl-aspartate
Source: PLoS One. 2020 Oct 1;15(10):e0239982. doi: 10.1371/journal.pone.0239982 (PMC7529218; doi:10.1371/journal.pone.0239982)

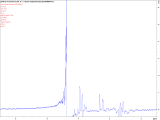

Supplement: S1 Data — (ZIP) [file pone.0239982.s002.zip › 3_NMR600_SISTEM_I_NAA_cream/78/pdata/1/thumb.png]

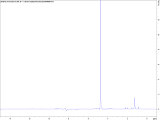

Supplement: S1 Data — (ZIP) [file pone.0239982.s002.zip › 3_NMR600_SISTEM_I_NAA_cream/80/pdata/1/thumb.png]

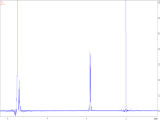

Supplement: S1 Data — (ZIP) [file pone.0239982.s002.zip › 1_NMR600_pH_data/F6_naa/1/pdata/1/thumb.png]

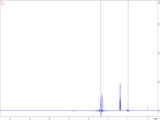

Supplement: S1 Data — (ZIP) [file pone.0239982.s002.zip › 1_NMR600_pH_data/F6_naa/2/pdata/1/thumb.png]

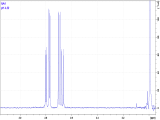

Supplement: S1 Data — (ZIP) [file pone.0239982.s002.zip › 1_NMR600_pH_data/F6_naa/3/pdata/1/thumb.png]

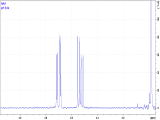

Supplement: S1 Data — (ZIP) [file pone.0239982.s002.zip › 1_NMR600_pH_data/F6_naa/4/pdata/1/thumb.png]

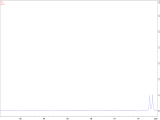

Supplement: S1 Data — (ZIP) [file pone.0239982.s002.zip › 1_NMR600_pH_data/F6_naa/5/pdata/1/thumb.png]

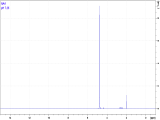

Supplement: S1 Data — (ZIP) [file pone.0239982.s002.zip › 1_NMR600_pH_data/F6_naa/6/pdata/1/thumb.png]

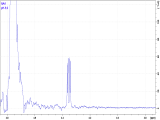

Supplement: S1 Data — (ZIP) [file pone.0239982.s002.zip › 1_NMR600_pH_data/F6_naa/7/pdata/1/thumb.png]

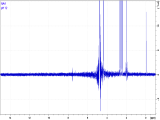

Supplement: S1 Data — (ZIP) [file pone.0239982.s002.zip › 1_NMR600_pH_data/F6_naa/8/pdata/1/thumb.png]

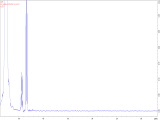

Supplement: S1 Data — (ZIP) [file pone.0239982.s002.zip › 1_NMR600_pH_data/FS2_lactate/1/pdata/1/thumb.png]

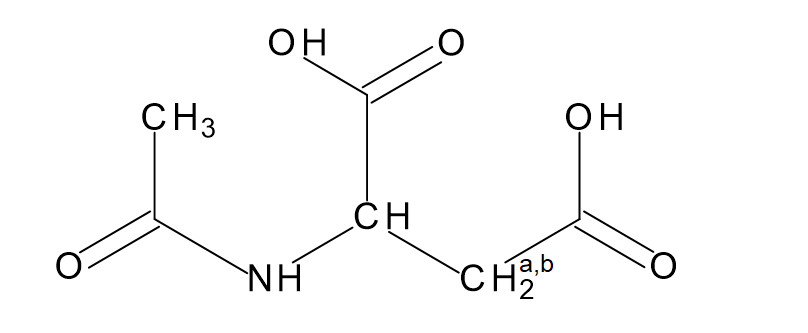

Supplement: S1 Scheme — The methyl protons (a, b) were used for SISTEM. The protons have J-coupling constant of 16–17 Hz with a chemical shift of 2.5–3 ppm depending on pH (SM). (TIF) [file pone.0239982.s003.tif]
